# Quality control of nonstop membrane proteins at the ER membrane and in the cytosol

**DOI:** 10.1038/srep30795

**Published:** 2016-08-02

**Authors:** Shunsuke Arakawa, Kaori Yunoki, Toshiaki Izawa, Yasushi Tamura, Shuh-ichi Nishikawa, Toshiya Endo

**Affiliations:** 1 Department of Chemistry, Graduate School of Science, Nagoya University, Chikusa-ku, Nagoya 464-8602, Japan; 2 Faculty of Life Sciences, Kyoto Sangyo University, Kamigamo-motoyama, Kita-ku, Kyoto 603-8555, Japan; 3 JST/CREST, Kyoto Sangyo University, Kamigamo-motoyama, Kita-ku, Kyoto 603-8555, Japan; 4 Research Center for Materials Science, Nagoya University, Chikusa-ku, Nagoya 464-8602, Japan; 5 Department of Material and Biological Chemistry, Faculty of Science, Yamagata University, 1-4-12 Kojirakawa-machi, Yamagata 990-8560, Japan; 6 Department of Biology, Faculty of Science, Niigata University, Nishi-ku, Niigata 950-2181, Japan

## Abstract

Since messenger RNAs without a stop codon (nonstop mRNAs) for organelle-targeted proteins and their translation products (nonstop proteins) generate clogged translocon channels as well as stalled ribosomes, cells have mechanisms to degrade nonstop mRNAs and nonstop proteins and to clear the translocons (e.g. the Sec61 complex) by release of nonstop proteins into the organellar lumen. Here we followed the fate of nonstop endoplasmic reticulum (ER) membrane proteins with different membrane topologies in yeast to evaluate the importance of the Ltn1-dependent cytosolic degradation and the Dom34-dependent release of the nonstop membrane proteins. Ltn1-dependent degradation differed for membrane proteins with different topologies and its failure did not affect ER protein import or cell growth. On the other hand, failure in the Dom34-dependent release of the nascent polypeptide from the ribosome led to the block of the Sec61 channel and resultant inhibition of other protein import into the ER caused cell growth defects. Therefore, the nascent chain release from the translation apparatus is more instrumental in clearance of the clogged ER translocon channel and thus maintenance of normal cellular functions.

Proteins are under the elaborate surveillance to maintain cellular protein homeostasis. Aberrant proteins could be generated upon translation as well as after their synthesis. When detected, aberrant nascent chains are subjected to repair, yet terminally deteriorated polypeptide chains are targeted to disposal by cellular degradation systems. mRNA lacking an in-frame stop codon called “nonstop mRNA” would generate aberrant “nonstop proteins” as well as accumulation of stalled ribosome-nascent chain (RNC) complexes. Generation of stalled RNC complexes calls for the nonstop-decay (NSD) pathway to degrade nonstop mRNAs and ribosomal quality control (RQC) factors to deal with nonstop proteins and recycle the stalled ribosomes^1^,^2^,^3^.

In the yeast NSD pathway, exosome is recruited by the GTPase Ski7, which enters the A site of stalled ribosomes and interact with the Ski2-Ski3-Ski8 complex, and degrades the 5′ fragment of nonstop mRNA in the 3′–>5′ direction^1^,^2^,^3^,^4^,^5^. The stalled RNC complexes are split into the 60S large and 40S small subunits by the complex of Dom34 (Pelota in mammals) and Hbs1^6^. Entry of Hbs1-GTP into the A site of stalled ribosomes allows its GTP hydrolysis and induces a conformational change in Dom34, which leads to recruitment of the ATPase Rli1 (ABCE1 in mammals) to ribosomes for driving subunit dissociation and recycling of the ribosomal subunits^7^,^8^,^9^. The nonstop cytosolic proteins are then ubiquitinated by Ltn1 (Listerin in mammals) associated with the RNC complex, and targeted to degradation by proteasomes^10^,^11^,^12^.

Although degradation of nonstop cytosolic proteins has been extensively studied^1^,^2^,^3^, fate of nonstop proteins targeted to organelles such as the endoplasmic reticulum (ER) and mitochondria was characterized only in a few studies^13^,^14^,^15^. Among them, we found that, when degradation of nonstop mRNAs does not work efficiently, nonstop proteins targeted to the ER or mitochondria occupy not only translating ribosomes but also translocons (translocators) in the organellar membranes^13^. The Dom34-Hbs1 complex acts on the stalled ribosomes to release stuck nonstop proteins into the organelle lumen, and if this release does not work efficiently, the protein flux into the organelle is blocked, resulting in defective cell growth. The cytosolic RQC pathway, which is important for the clearance of nonstop proteins in the cytosol, may not operate sufficiently for those nonstop proteins targeted to the ER and mitochondria. The reason for such escape of certain nonstop organelle-targeted proteins from the cytosolic RQC pathway could be the limited exposure of the segments for cytosolic ubiquitination, although recent studies showed that such ubiquitination may take place under some conditions^14^,^15^. Then, a question arises what the fate of nonstop organellar membrane proteins are, which contain a segment(s) exposed to the cytosol for possible ubiquitination by the cytosolic RQC pathway.

In the present study, we asked which of the pathways, the cytosolic Ltn1-dependent degradation and the Dom34-Hbs1 dependent release into the ER lumen, nonstop ER membrane proteins can be targeted to. We thus followed the fate of nonstop membrane proteins at the ER membrane with different membrane topologies, and found that the latter pathway is more important for the quality control of nonstop membrane proteins at the ER membrane.

## Results

### Nonstop membrane proteins can be degraded by proteasome in the cytosol

**Figure 1:**
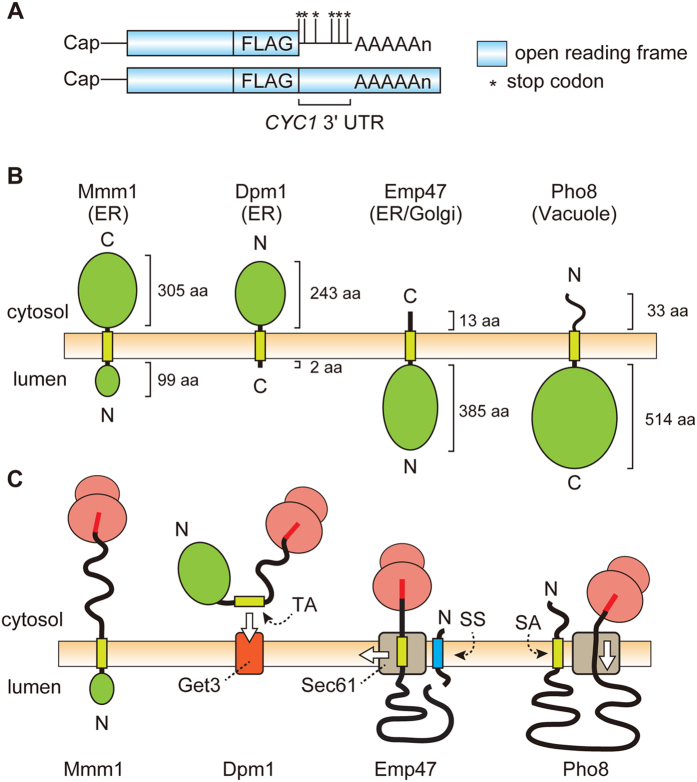
mRNA diagrams, membrane topologies, and possible engagement with the transclocon of the ER membrane proteins used in this study. (**A**) Schematic diagrams of mRNAs and (**B**) membrane topologies of the ER membrane proteins studied here. Final destinations of the membrane proteins are indicated in parentheses. (**C**) Possible engagement of nonstop ER membrane proteins with the Sec61 translocon channel. Mmm1: A protein that follows the N-terminus translocation pathway mediated by the TM segment with the relatively short luminal segment and a large C-terminal cytosolic domain. Dpm1: A tail-anchor protein, having the tail-anchor (TA) membrane targeting sequence at the C-terminus, whose membrane integration is mediated by the Get3 machinery. Emp47: A protein whose translocation is initiated by the cleavable signal sequence (SS) to generate the large luminal domain, but halted by the TM segment near the C-terminus. Pho8: A protein with a large luminal domain, including the ribosome-engaging C-terminal end, that is translocated by the type II signal-anchor (SA) sequence.

We first asked if nonstop ER membrane proteins are under the surveillance for cytosolic ubiquitination followed by proteasomal degradation. Since ubiquitination requires exposure of a substrate segment to the cytosol, we suppose that the cytosolic RQC for nonstop ER membrane proteins, if any, depends on their membrane topologies. We thus chose four different membrane proteins with a single transmembrane (TM) segment, Mmm1^16^, Dpm1^17^, Emp47^18^, and Pho8^19^, with different membrane topologies and made their nonstop variants by constructing corresponding genes lacking a stop codon, but followed by a DNA segment for the FLAG epitope tag, translated *CYC1* 3′-UTR and Lys repeats encoded by the poly(A) tail in mRNA (Fig. 1A). Membrane topologies of Mmm1, Dpm1, Emp47, and Pho8 and expected configurations with the membrane, ribosome, and translocon for their nonstop variants are shown in Fig. 1B,C, respectively. Mmm1 follows the N-terminus translocation pathway mediated by the TM segment with the relatively short luminal segment, and has a large C-terminal cytosolic domain, which should separate the translation completing ribosome from the translocon. Therefore this protein may be analogous to soluble cytosolic proteins in the context of the nonstop effects. Dpm1 is a tail-anchor protein with the membrane targeting TM sequence at the C-terminus. Membrane integration of Dpm1 is mediated by the Get3 pathway, which is only possible after complete termination of translation. Translocation of Emp47 is initiated by the cleavable signal sequence to produce the large luminal domain, but halted by the TM segment, which functions as a “stop-transfer” sequence. The TM segment followed by a very short C-terminal, cytosolic tail should eventually be partitioned into the lipid phase of the ER membrane by laterally exiting from the translocon channel. Pho8 has the large luminal domain and is translocated by the type II signal-anchor sequence. Therefore, this protein is analogous to the soluble ER protein in the context of the nonstop effects, and what matters for the nonstop variant is the translocation of the C-terminal end.

**Figure 2:**
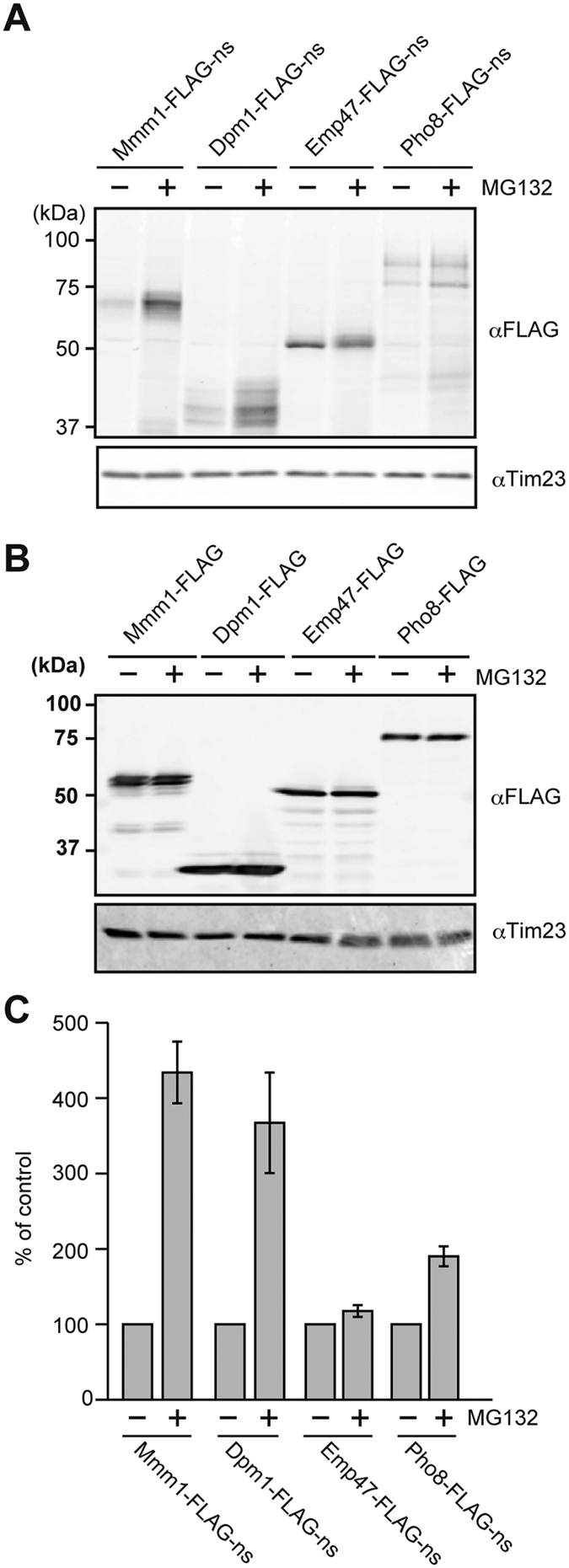
Nonstop ER membrane proteins are differently affected by the proteasomal inhibitor MG132. (**A**,**B**) Whole cell extracts prepared from yeast cells expressing the indicated nonstop (**A**) or stop (**B**) membrane proteins from the *GPD1* promoter with or without MG132 treatments (100 μM for 1.5 h). (**C**) Quantification of the levels of nonstop membrane proteins with and without MG132 treatments. The protein levels without MG132 treatments were set to 100%. Error bars represent SD (*n* = 3).

We prepared whole cell extracts from the cells expressing Mmm1-FLAG, Dpm1-FLAG, Emp47-FLAG, or Pho8-FLAG and from those expressing their nonstop variants in the presence or the absence of a proteasome inhibitor MG132. When we compared their protein levels by immnoblotting with the anti-FLAG antibody (Fig. 2A,B), we observed significant stabilization of Mmm1-FLAG-ns and Dpm1-FLAG-ns and moderate stabilization of Pho8-FLAG-ns by MG132 while the level of Emp47-FLAG-ns was not increased by MG132 (Fig. 2A,C). MG132 did not alter the levels of corresponding normally translated proteins, Mmm1-FLAG, Dpm1-FLAG, Emp47-FLAG, or Pho8-FLAG (Fig. 2B). These results suggest that the nonstop membrane proteins, Mmm1-FLAG-ns, Dpm1-FLAG-ns, or Pho8-FLAG-ns, but not Emp47-FLAG-ns, can be targeted to disposal by the cytosolic RQC involving proteasomal degradation.

### Expression of nonstop membrane proteins causes defective cell growth in the absence of Dom34 or Ltn1 when NSD is blocked

**Figure 3:**
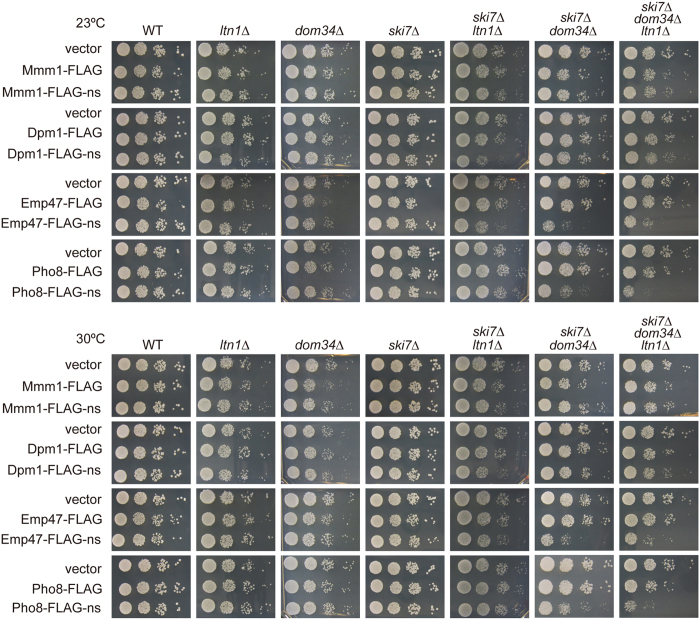
Effects of expression of stop or nonstop ER membrane proteins on yeast cell growth. Serial dilutions with 10-fold increments of the indicated yeast cells with or without expression of the indicated nonstop or stop membrane proteins from the *GPD1* promoter were spotted onto SCD-Ura plates and cultivated at 23 °C or 30 °C for 3 or 2 days, respectively.

Previously, we found that expression of nonstop secretory-pathway proteins like vacuolar carboxypeptidase Y-ns (CPY-ns) leads to defective cell growth when quality control factors for nonstop mRNA such as Ski7 and those for stalled ribosomes such as Dom34 and Hbs1 are absent^13^. This is because, when the nonstop protein level increases due to defective NSD, nonstop ER-targeted proteins occupy the translocon at the ER membrane, thereby blocking influx of other secretory-pathway proteins. We thus examined whether expression of nonstop membrane proteins also affects cell growth when quality control factors for nonstop proteins and/or nonstop mRNA are absent (Fig. 3).

Since Ski7 mediates degradation of nonstop mRNA, the *ski7*Δ mutation leads to increased levels of nonstop proteins^2^,^3^,^4^,^5^. Dom34 mediates splitting of the large (60 S) and small (40 S) ribosomal subunits for NSD of nonstop mRNA^6^ and for release and/or ubiquitination of nonstop nascent chains^9^,^11^. Ltn1, an E3 ubiquitin ligase in the RQC pathway, ubiquitinates nonstop nascent chains for proteasomal degradation in the cytosol^10^,^11^. We previously found that expression of a non-stop ER-targeted soluble protein CPY-FLAG-ns, but not its signal-peptide lacking variant ΔSP-CPY-FLAG-ns, causes defective cell growth in *dom34*Δ*ski7*Δ cells^13^. However, we observed no growth defects for *dom34*Δ*ltn1*Δ cells upon expression of CPY-FLAG-ns or ΔSP-CPY-FLAG-ns (Fig. S1A,C). Expression of Emp47-FLAG-ns or Pho8-FLAG-ns but not Emp47-FLAG and Pho8-FLAG caused the defective growth of *ski7∆dom34∆* cells although it did not affect those of wild-type and single deletion mutant, *ski7∆* or *dom34∆*, cells. In contrast, expression of Dpm1-FLAG-ns, and Emp47-FLAG-ns slightly, led to defective cell growth when the *ltn1∆* mutation was combined with the *ski7∆* or *ski7∆dom34∆* mutation. Mmm1-FLAG-ns expression did not affect cell growth significantly with any combination of the mutations *ltn1*Δ, *dom34*Δ, and *ski7*Δ. These results suggest that absence of Dom34 and/or Ltn1 together with deletion of *SKI7* also affect cell growth upon expression of some nonstop membrane proteins.

### Ltn1 mediates degradation of nonstop membrane proteins while Dom34 prevents their block of the ER protein import

To gain more insights into the roles of Dom34 and Ltn1 in the quality control of nonstop membrane proteins, we analyzed the levels of nonstop membrane proteins in cells lacking Ski7, Dom34 and/or Ltn1. First, we found that, while protein levels of both ER-targeted CPY-FLAG-ns and cytosolic ΔSP-CPY-FLAG-ns increased in *ski7∆* cells as compared with wild-type cells^13^, that of ΔSP-CPY-FLAG-ns, but not of CPY-FLAG-ns, increased in *ski7∆ltn1∆* cells as compared with *ski7∆* cells (Fig. S1B,D). This suggests that ΔSP-CPY-FLAG-ns, not CPY-FLAG-ns, is subject to Ltn1-dependent RQC in the cytosol.

**Figure 4:**
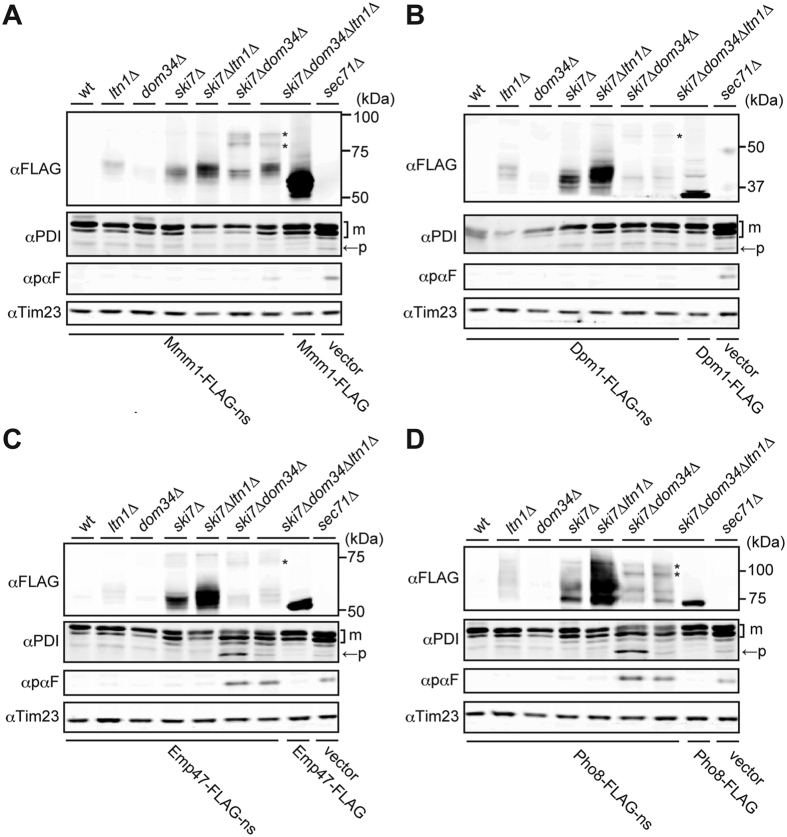
Roles of Ltn1 in protein levels and of Dom34 in the ER protein import upon expression of nonstop ER membrane proteins. Whole cell extracts prepared from the indicated yeast cells with expression of the indicated nonstop or stop membrane proteins from the *GPD1* promoter were analyzed by SDS-PAGE and immunoblotting using the indicated antibodies. Asterisks indicate tRNA attached forms. m, mature protein; p, precursor form.

Next, we analyzed the levels of nonstop membrane proteins in cells with different combinations of *ski7∆*, *dom34∆*, and *ltn1∆*. Like nonstop ER-targeted soluble proteins, steady state levels of nonstop membrane proteins significantly increased in *ski7∆* cells, as compared with wild-type cells (Fig. 4). Importantly, the *ltn1∆* mutation stabilized all the nonstop membrane proteins (Fig. 4). Protein levels were further increased when the *ski7∆* and *ltn1∆* mutations were combined, indicating that Ltn1 is involved in degradation of not only cytosolic nonstop proteins but also nonstop membrane proteins. On the other hand, the *dom34*∆ mutation decreased the protein levels of the nonstop-membrane proteins in *ski7*∆ cells. This is most likely due to suppressed turnover of the ribosomes available for translation of nonstop mRNA because loss of Dom34 leads to defective ribosomal recycling, thereby accumulating stalled RNCs. This interpretation is consistent with the observation that peptidyl-tRNA forms of nonstop membrane proteins, which were sensitive to RNase digestion, were detected in *ski7∆dom34∆* and *ski7∆dom34∆ltn1∆* cells (Fig. 4, asterisks; Fig. S2).

Since the protein levels of the examined nonstop membrane proteins (Fig. 4) are not correlated well with the growth defects (Fig. 3), we reasoned that, like expression of CPY-ns, expression of Emp47-FLAG-ns or Pho8-FLAG-ns compromises the protein flux into the ER in *ski7∆dom34∆* cells. Indeed, when Emp47-FLAG-ns or Pho8-FLAG-ns were expressed in *ski7∆dom34∆* and *ski7∆dom34∆ltn1∆* cells, precursor forms of protein disulfide isomerase (PDI) and α-mating factor (αF) were accumulated (Fig. 4), which is similar to the case of *sec71*Δ mutant cells^20^ with defective ER translocon functions. These results suggest that Emp47-FLAG-ns and Pho8-FLAG-ns block the import of other secretory-pathway proteins into the ER at the level of the ER translocon, which would be harmful to the normal cellular processes.

### Mmm1-FLAG-ns, Emp47-FLAG-ns, and Pho8-FLAG-ns, but not Dpm1-FLAG-ns, are correctly targeted to the ER membrane

**Figure 5:**
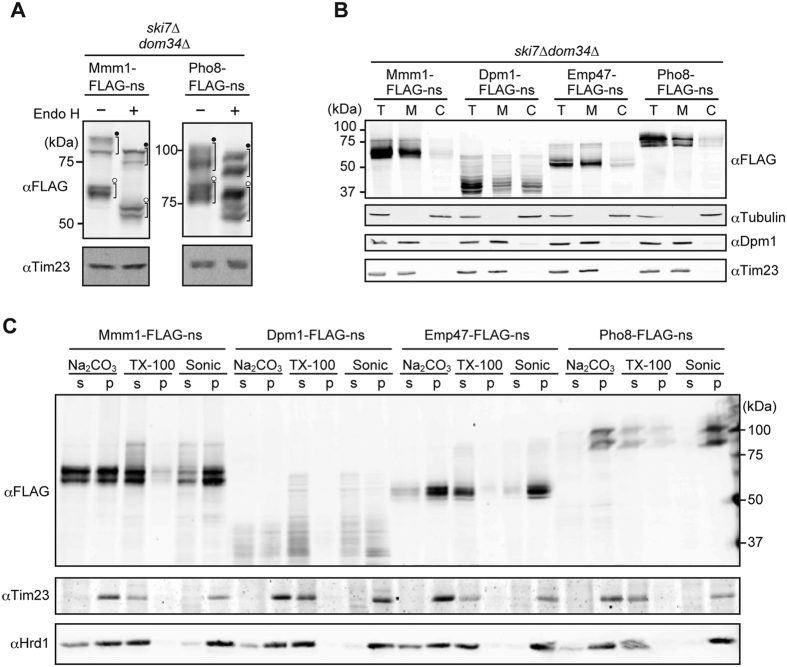
Cellular localization and membrane association of nonstop ER membrane proteins. (**A**) Whole cell extracts prepared from *ski7∆dom34∆* cells expressing Mmm1-FLAG-ns or Pho8-FLAG-ns from the *GPD1* promoter were incubated with or without Endo H (500 unit/ml) at 37 °C for 40 min, and analyzed by SDS-PAGE and immunoblotting. Closed dots and open dots indicate tRNA-attached and tRNA-dissociated forms, respectively. (**B**) *ski7∆dom34∆* cells expressing the indicated nonstop membrane proteins from the *GPD1* promoter were fractionated by differential centrifugations. T, total; M, High-speed precipitate containing membrane fractions; (**C**) High-speed supernatant containing cytosolic fractions. (**C**) The membrane fractions prepared in (**B**) were treated with Na_2_CO_3_ at pH 9.4 or Triton X-100 (TX-100), or sonicated (sonic), and then subjected to ultracentrifugation. The resulting supernatant (s) and precipitate (p) were analyzed by SDS-PAGE and immunoblotting using the indicated antibodies.

We next asked if the nonstop membrane proteins are correctly targeted to the ER. For this purpose, we took advantage of the fact that Mmm1-FLAG-ns and Pho8-FLAG-ns have potential *N*-glycosylation sites in the ER luminal domains. Whole cell extracts were prepared from *ski7∆dom34∆* cells expressing Mmm1-FLAG-ns or Pho8-FLAG-ns and subjected to the treatment with endoglycosidase H (Endo H), which cleaves *N*-glycans, followed by immunoblotting with the anti-FLAG antibody. Mmm1-FLAG-ns and Pho8-FLAG-ns showed multiple bands arising from the forms with and without attachment of tRNA (Fig. 5A). All these bands shifted to the lower molecular weight regions after Endo H treatments while the bands from a mitochondrial protein Tim23 without any glycosylation did not (Fig. 5A). Therefore the ER luminal domains of Mmm1-FLAG-ns and Pho8-FLAG-ns are normally imported into the ER to receive *N*-glycosylation.

We then performed cell fractionation to assess membrane association of the nonstop membrane proteins (Fig. 5B). Mmm1-FLAG-ns, Pho8-FLAG-ns, and Emp47-FLAG-ns were collected in the membrane fractions, like a mitochondrial protein Tim23 and an ER protein, endogenous Dpm1, suggesting that Emp47-FLAG-ns as well as Mmm1-FLAG-ns and Pho8-FLAG-ns was targeted to the ER membrane. On the other hand, Dpm1-FLAG-ns was recovered in both the membrane and high-speed supernatant fractions. This suggests that the Dpm1-FLAG-ns targeting to the ER was compromised, probably due to inefficient recognition by Get3 of the GET system in the presence of the attached ribosome^21^,^22^.

We probed insertion of the nonstop membrane proteins into the membrane by alkaline extraction, Triton X-100 treatment, and sonication (Fig. 5C). The integral membrane proteins, Tim23 in the mitochondrial inner membrane and Hrd1 in the ER membrane, showed resistance to alkaline extraction with Na_2_CO_2_, were solubilized with Triton X-100, and were recovered in the pellet fraction by ultracentrifugation after sonication. Mmm1-FLAG-ns, Pho8-FLAG-ns, and Emp47-FLAG-ns, not Dpm1-FLAG-ns, behaved like those control integral membrane proteins. This again indicates that, while Mmm1-FLAG-ns, Pho8-FLAG-ns, and Emp47-FLAG-ns were targeted to and inserted into the ER membrane or got stuck at the integral membrane protein complex, the Sec61 complex, targeting of Dpm1-FLAG-ns to the ER was partly impaired.

### Pho8-FLAG-ns and Emp47-FLAG-ns block the ER translocon channel

**Figure 6:**
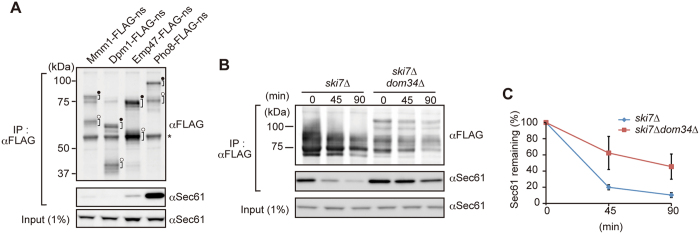
Pho8-FLAG-ns is stably associated with Sec61. (**A**) Cell extracts were prepared from *ski7∆dom34∆* cells expressing the indicated nonstop proteins from the *GPD1* promoter by vortexing with glass beads, solubilized with 1.0% digitonin and then subjected to co-immunoprecipitation using anti-FLAG agarose beads. The immunoprecipitated proteins were analyzed by SDS-PAGE and immunoblotting. Asterisks, closed dots, and open dots indicate IgG bands, tRNA-attached forms, and tRNA-dissociated forms, respectively. (**B**,**C**) The indicated cells expressing Pho8-FLAG-ns from the *GPD1* promoter were harvested at the indicated times after the addition of 200 μg/ml of cycloheximide. The cell extracts were prepared and analyzed as in (**A**). The amounts of Sec61 for the cells right after the addition of cycloheximide were set to 100%. Error bars represent SD (*n* = 3).

We previously found that CPY-ns gets stuck in the ER translocon channel due to its attached ribosome, which causes impaired ER protein import^13^. Indeed, accumulation of precursor forms of PDI and αF upon expression of Emp47-FLAG-ns and Pho8-FLAG-ns, not of Mmm1-FLAG-ns or Dpm1-FLAG-ns, in *ski7∆dom34∆* and *ski7∆dom34∆ltn1∆* cells suggests that Emp47-FLAG-ns and Pho8-FLAG-ns get stuck at the ER translocon when the ribosome splitting by Dom34 is impaired, resulting in the block of the import of other secretory-pathway proteins into the ER (Fig. 4). We thus tested if nonstop membrane proteins get stuck in the Sec61 channel by co-immunoprecipitation experiments. Whole cell extracts from *ski7∆dom34∆* cells expressing Mmm1-FLAG-ns, Dpm1-FLAG-ns, Pho8-FLAG-ns or Emp47-FLAG-ns were solubilized with digitonin, and were subjected to co-immunoprecipitation using anti-FLAG agarose beads (Fig. 6A). Interestingly, while a significant amount of Sec61 was co-immunoprecipitated with Pho8-FLAG-ns, Emp47-FLAG-ns co-immunoprecipitated only a minute amount of Sec61. Neither Mmm1-FLAG-ns nor Dpm1-FLAG-ns co-immunoprecipitated Sec61.

Why does expression of Pho8-FLAG-ns and Emp47-FLAG-ns, but not Mmm1-FLAG-ns or Dpm1-FLAG-ns, block the protein flux into the ER, resulting in growth defects in *ski7∆dom34∆* but not in *ski7∆* cells (Fig. 3)? Like the case of CPY-FLAG-ns^13^, association of Pho8-FLAG-ns with Sec61 persisted after stopping protein synthesis by cycloheximide in *ski7∆dom34∆* cells as compared with the case in *ski7*∆ cells (Fig. 6B,C). Therefore, the nonstop membrane proteins like Pho8-FLAG-ns without a TM segment near the ribosome-engaging C-terminus stably occupy Sec61 channels for a longer time in *ski7∆dom34∆* cells than in *ski7∆* cells, so that the protein flux into the ER is significantly blocked. On the other hand, the C-terminal TM segment of Emp47-FLAG-ns could be laterally released from the translocon to be partitioned into the lipid phase of the ER membrane. However, the lateral exit of the TM segment from the translocon usually requires disengagement of the ribosome as a cue^23^. Therefore C-terminal attachment of the stalled ribosome in the absence of Dom34 may well retard the lateral release step of the TM segment of Emp47-FLAG-ns, resulting in the clogged translocon, as well (Fig. 1C). Notably, solubilization of the ER membrane would destabilize such ribosome-translocon interactions, thereby allowing the lateral leave of the TM segment of Emp47-FLAG-ns from the translocon, as indicated by only a minute amount of Sec61 co-immunoprecipitated with Emp47-FLAG-ns (Fig. 6A).

## Discussion

In the present study, we addressed the questions of how ER-targeted membrane proteins are handled by the ribosome-based cellular protein quality control mechanisms when their translation encounters aberrant situations such as a lack of a stop codon. To obtain basic knowledge of this previously unasked question, we focused on nonstop single-membrane-spanning proteins with different membrane topologies and different modes of engagement with the membrane insertion machineries of the cell. We found that deletion of *DOM34* together with *SKI7* leads to growth defects upon expression of Emp47-FLAG-ns or Pho8-FLAG-ns, yet deletion of *LTN1* together with *SKI7* gave no or only a slight effect on cell growth upon expression of those proteins. Since Pho8-FLAG-ns is completely resistant against alkaline treatments in *ski7∆dom34∆* cells (Fig. 5C), its TM segment appears to have already been released laterally from the Sec61 channel to the ER membrane. However, due to the membrane topology and C-terminal association with the ribosome (Fig. 1C), the C-terminal hydrophilic segment after the TM segment of Pho8-FLAG-ns may well stay inside the Sec61 channel in *ski7∆dom34∆* cells. Indeed, Pho8-FLAG-ns is associated with Sec61 in *ski7∆dom34∆* cells (Fig. 6A) and this association is stabilized when Dom34 is absent in the *ski7*Δ background (Fig. 6B,C). The prolonged residence of the C-terminal segment of Pho8-FLAG-ns in the Sec61 channel would severely prevent the ER import of other secretory-pathway proteins (Fig. 4D). Such a block of the protein flux into the ER results in cell growth defects (Fig. 3) as observed for nonstop soluble proteins in the secretory pathways^13^.

Translocation of Emp47-FLAG-ns starts from the cleavable signal sequence at the N-terminus and subsequent translocation that generate the large luminal domain is halted by the TM segment as a stop transfer sequence. This TM segment near the C-terminus could be released laterally from the Sec61 translocon channel, but such a lateral exit requires disengagement of the ribosome from the translocon^23^, which cannot take place for the stalled ribosome attached to Emp47-FLAG-ns with the TM segment followed by a short C-terminal segment. This suggests that Emp47-FLAG-ns also occupied the Sec61 channel at the step of lateral exit in *ski7∆dom34∆* cells, although direct Sec61 association with Emp47-FLAG-ns was only slightly retained after membrane solubilization. This occupation of the translocon channel by the TM segment of Emp47-FLAG-ns appears sufficient to block the ER protein import in general (Fig. 4C) and to result in growth defects (Fig. 3). Indeed, the attached ribosome may well sterically interfere with the secretory protein pathway because of its large size. In addition, the recent structural studies on the bacterial and ER Sec translocons show that the lateral exit region is also used for initial recognition of the signal sequence^24^,^25^, suggesting that the stuck TM segment of Emp47 at the lateral gate of the Se61 channel would block translocation of other secretory pathway proteins through the channel.

In contrast to Emp47-FLAG-ns, Mmm1-FLAG-ns with the large C-terminal domain in the cytosol does not interact with Sec61 at all (Fig. 6A), although it is moderately resistant to alkaline extraction in *ski7∆dom34∆* cells (Fig. 5C). Mmm1-FLAG-ns is likely released efficiently from the Sec61 channel into the ER membrane, probably because the TM segment of Mmm1-FLAG-ns functions as a type I signal anchor segment and interactions between the attached ribosome and translocon did not last long due to the presence of the large folded C-terminal domain (Fig. 1C). Mmm1-FLAG-ns thus does not affect the influx of secretory-pathway proteins into the ER even in the absence of Dom34.

Dpm1, a tail anchor protein in the ER membrane, is targeted to the ER membrane by the GET system and inserted into the ER membrane by C-terminal tail-anchor TM segment in a post-translational manner without using the Sec61 translocon^21^,^22^. However for Dpm1-FLAG-ns, C-terminal attachment of the ribosome would hamper correct recognition of the targeting signal by Get3 of the GET system. Since binding to the internal TM segment is more favored by the signal recognition particle (SRP) than Get3^21^, Get3 binding to the TM segment of Dpm1-FLAG-ns, which is not positioned at the C-terminus, may be strongly competed by the SRP. Dpm1-FLAG-ns was thus only partly associated with membranes (Fig. 5B) and was not stably associated with the Sec61 channel in *ski7∆dom34∆* cells (Fig. 6A). Dpm1-FLAG-ns hence did not cause growth defects in *ski7∆dom34∆* cells arising from the intervention in the ER protein import via the Sec61 translocon.

What is the role of Dom34 in the quality control of nonstop membrane proteins? We previously found that nonstop soluble ER-targeted proteins including CPY-ns can be released into the ER lumen in a Dom34- and Hbs1-dependent manner, which clears the clogged Sec61 channel^13^. We also found that the mitochondrial translocator channel clogged by nonstop mitochondrial matrix proteins was also cleared by the actions of Dom34 and Hbs1^13^. Dom34 and Hbs1 cause ribosomal splitting, which facilitates the luminal release of nonstop proteins upon hydrolysis of a peptidyl-tRNA linkage. The C-terminal segment of Pho8-FLAG-ns, which occupies the Sec61 channel, is likely released from the Sec61 channel into the ER lumen by the similar mechanism by Dom34 and Hbs1, resulting in the clearance of the otherwise clogged Sec61 channel. Occupancy of the Sec61 complex by RNC containing Emp47-FLAG-ns that inhibits other protein import into the ER may be relieved by the release of the C-terminal segment from the stalled ribosome by the action of Dom34 and Hbs1, which would trigger the lateral release of the stuck TM segment from the Sec61 channel.

Interestingly in the absence of NSD, the nonstop membrane proteins studied here were all stabilized by depletion of cytosolic ubiquitin ligase Ltn1 (Fig. 4). This indicates that these nonstop membrane proteins have cytosolically exposed segments for ubiquitination by Ltn1. If these nonstop membrane proteins have a cytosolically exposed C-terminal segments, their Ltn1-mediated ubiquitination can be explained easily because such segments will engage with the stalled ribosome in the case of non-stop variants. Indeed, this appears to be the case for Mmm1-FLAG-ns having a large C-terminal cytosolic domain and Emp47-FLAG-ns having the cytosolic, 13-residue tail intrinsically, which contains a lysine residue and is further followed by an extension of about 60 residues until reaching the exit gate of the ribosomal tunnel (Fig. 1B,C). In contrast, Pho8-FLAG-ns has the cytosolically exposed N-terminus, which is unlikely to be a ubiquitination target because of its position distal to the stalled ribosome. Pho8-FLAG-ns has difficulties in having its C-terminus threading through the translocon because of the obstruction by the stalled ribosome. This is analogous to the non-stop situation of soluble lumenal proteins studied previously, in which the ubiquitination site is assumed to have access to the cytosol only through the translocon-ribosome junction^14^,^15^.

Dpm1-FLAG-ns primarily stays in the cytosol for ubiquitination. On the other hand, while a proteasomal inhibitor MG132 stabilized Mmm1-FLAG-ns and Dpm1-FLAG-ns significantly, it stabilized Emp47-FLAG-ns and Pho8-FLAG-ns only slightly (Fig. 2), suggesting that the inhibitory effect of MG132 may be partial under the present conditions. Dom34 appears to be required for Ltn1-depndent degradation (Fig. 4), but more importantly, this Ltn1- and Dom34-dependent degradation of nonstop membrane proteins is not correlated well with the block of the ER protein import (Fig. 4), which most likely causes the cell growth defects like Sec translocon mutants^26^. Therefore occupation of the ER translocon channel, not of the translating ribosomes, appears to give a crucial damage to normal cellular functions.

What is the role of Ltn1-dependent degradation of nonstop membrane proteins? Since deletion of *LTN1* in the absence of the NSD caused defective growth for only the nonstop tail-anchor protein, Dpm1-FLAG-ns, Ltn1 dependent degradation facilitated by Dom34 and Hbs1 may be important for clearance of nonstop membrane proteins from the GET system, the number of which may be more limited than abundant ribosomes in the cell. Recently, nonstop ER-targeted soluble proteins that get stuck at the Sec61 complex were found to be ubiquitinated by Ltn1/Listerin^14^,^15^. When a nonstop VHP fusion protein containing a signal sequence and luminal folded VHP domain followed by a long unstructured segment was imported into microsomes *in vitro*, the fusion protein gets stuck in the Sec61 channel, but the C-terminal unstructured segment may loop out through the cytosolic gap between the ribosome and Sec61, and is ubiquitinated^14^. Crowder *et al.* observed that nonstop CPY followed by a tandem repeat of protein A domains was significantly stabilized by the *LTN1* deletion and MG132 *in vivo*^15^. However, we did not observe such stabilization of CPY-FLAG-ns in *ski7*Δ*ltn1*Δ cells as compared with *ski7*Δ cells (Fig. S1). Since CPY is imported into the ER partly by a post-translational manner^27^, the observed stabilization by MG132 or *LTN1* deletion probably reflects the hampered post-translational import of the nonstop CPY fusion protein due to the presence of tightly folded protein A domains, which results in targeting to the proteasomal degradation pathway. Actually we also observed partial stabilization of CPY-ns without a protein A domain by MG132^13^, which suggests that a fraction of CPY-ns molecules, even without a protein A domain, could be targeted to the cytosolic RQC pathway likely before engagement with the ER translocon. MG132 does not stabilize the nonstop CPY variant with ER-targeting signal sequence replaced with that from Ost1, which is imported into the ER by a co-translational, not post-translational, manner^15^. Nevertheless, physiological relevance of ubiqutination of nonstop proteins at the ER translocon still remains unclear as the glycosylated luminal domain of the stuck nonstop proteins may not be translocated back through the narrow Sec61 channel to the cytosol for proteasomal degradation^28^,^29^. Therefore more studies need to be done to obtain a comprehensive view on the fate of nonstop proteins at the organellar membranes.

## Materials and Methods

### Yeast strains and plasmids

The *CgTRP1* gene cassette with homologous regions to the 5′ and 3′-UTR of the *LTN1* gene was amplified from pCgTRP1^30^ using primers (ltn1-KO-F: 5′-TCG TTT GGT GGA ATC AAT ACA TTT CAA CAG TAT AAC ACA GGT TGT AAA ACG ACG GCC AGT-3′ and ltn1-KO-R: 5′-GAC GCC ACT AAG AGT CTG GTT CAG TTT TGT GTA CTC AGA TCA CAG GAA ACA GC TAT GAC C-3′) and was introduced into HNY2, IZY4 and IZY15 strains^13^ to obtain *ltn1*∆, *ski7*∆*ltn1*∆ and *ski7*∆*dom34*∆*ltn1*∆, respectively. To express C-terminally FLAG-tagged proteins or the nonstop variants, the gene encoding Mmm1, Dpm1, Emp47 or Pho8 was PCR-amplified using primers (MMM1-inF F1: 5′-GGA TTC TAG AAC TAG TAT GAC TGA TAG TGA GAA TGA-3′ and MMM1-inF R1: 5′-GCT TGA TAT CGA ATT CTA ACT CTG TAG GCT TTT CTT-3′; DPM1-inF F1: 5′- GGA TTC TAG AAC TAG TAT GAG CAT CGA ATA CTC TGT-3′ and DPM1-inF R1: 5′-GCT TGA TAT CGA ATT CAA AGA CCA AAT GGT ATA GCT-3′; EMP47-inF F1 : 5′-GGA TTC TAG AAC TAG TAT GAT GAT GTT AAT TAC TAT-3′ and EMP47-inF R1: 5′-GCT TGA TAT CGA ATT CTA GCA GTT TGG TCT TTA TGA-3′; Pho8-inF F1: 5′-GGA TTC TAG AAC TAG TAT GAT GAC TCA CAC ATT ACC-3′ and Pho8-inF R1:5′-GCT TGA TAT CGA ATT CGT TGG TCA ACT CAT GGT AGT-3′) and ligated into SpeI/EcoRI-cut vector by In-fusion cloning (Takara/Clontech), pIZ7 and pIZNF1, respectively^13^.

### Co-immunoprecipitation

Yeast cells were cultivated in SCD-Ura media at 30 °C for over 12 h until OD_600_ reaches 1.0. Fifty OD_600_ units of yeast cells (50 ml culture of OD_600_ = 1.0) were precipitated and suspended in ice-cold 10 mM NaN_3_. For the cycloheximide chase and co-immunoprecipitation assay, freshly prepared cycloheximide solution was added to the cell culture to 200 μg/ml. The yeast cells were suspended in 500 μl of buffer (20 mM Tris-HCl, pH 6.8, 150 mM NaCl, 1 mM phenylmethylsulfonylfluoride, protease inhibitor cocktail) with 200 μl of glass beads and were vortexed 8 times for 30 sec with an interval of 30 sec on ice. After removal of glass beads and unbroken cells by centrifugation at 1,000 × *g* for 5 min, membranes in the supernatant were solubilized with 1% digitonin. After removal of insoluble debris by centrifugation at 22,000 × *g* for 20 min, anti-FLAG M2 agarose was added to the supernatant, which was gently rotated for 1.5 h at 4 °C. After washing 4 times with wash buffer 1 (0.1% digitonin, 20 mM Tris-HCl, pH 6.8, 150 mM NaCl) and once with wash buffer 2 (20 mM Tris-HCl, pH 6.8, 150 mM NaCl), the anti-FLAG agarose beads were boiled in SDS-PAGE sample buffer at 95 °C for 5 min. Eluted proteins were analyzed by SDS-PAGE or Nu-PAGE (Life Technologies) followed by immunoblotting.

### Cell fractionation

Yeast cells were cultivated in SCD-Ura media at 30 °C for over 12 h until the OD_600_ reaches 1.0. Twenty OD_600_ units of yeast cells (20 ml culture of OD_600_ = 1.0) were incubated in alkaline buffer (100 mM Tris-HCl pH 9.6, 10 mM dithiothreitol) at room temperature for 15 min. Then, the cells were washed once with 1.2 M sorbitol buffer (1.2 M sorbitol, 20 mM Tris-HCl, pH 7.4) and incubated with 1.2 M sorbitol buffer containing 2 unit/ml Zymolyase 20 T at 30 °C fro 45 min. The resulting spheroplasts were suspended in 200 μl of 0.7 M Sorbitol buffer (0.7 M sorbitol, 20 mM MOPS, pH 7.2, 5 mM MgCl_2_, 1 mM phenylmethylsulfonylfluoride, protease inhibitor cocktail) containing 200 μl of glass beads and were vortexed twice for 1 min with an interval of 1 min on ice. After removal of glass beads and unbroken cells by centrifugation at 1,000 × *g* for 5 min at 4 °C, ~300 μl of supernatant fraction was collected. 150 μl out of 300 μl of the supernatant was used as total lysate. Another 150 μl of the supernatant was subjected to centrifugation at 15,000 × *g* for 15 min at 4 °C and the resulting pellet and supernatant were used as membrane and cytosol fractions, respectively. All the samples (total lysate, membrane and cytosol fractions) were solubilized in 225 μl of SDS sample buffer.

## Additional Information

**How to cite this article**: Arakawa, S. *et al.* Quality control of nonstop membrane proteins at the ER membrane and in the cytosol. *Sci. Rep.* **6**, 30795; doi: 10.1038/srep30795 (2016).

## Supplementary Material

Supplementary Information

## References

[b1] Inada T. Quality control systems for aberrant mRNAs induced by aberrant translation elongation and termination. Biochim Biophys Acta 1829, 634–642 (2013). 23416749 10.1016/j.bbagrm.2013.02.004

[b2] Lykke-Andersen J. & Bennett E. J. Protecting the proteome: eukaryotic cotranslocational quality control pathways. J Cell Biol. 204, 467–476 (2014). 24535822 10.1083/jcb.201311103 PMC3926952

[b3] Shoemaker C. J. & Green R. Translation drives mRNA quality control. Nat Struct Mol Biol. 19, 594–601 (2012). 22664987 10.1038/nsmb.2301 PMC4299859

[b4] Frischmeyer P. A. *et al.* An mRNA surveillance mechanism that eliminates transcripts lacking termination codons. Science 295, 2258–2261 (2002). 11910109 10.1126/science.1067338

[b5] van Hoof A., Frischmeyer P. A., Dietz H. C. & Parker R. Exosome- mediated recognition and degradation of mRNAs lacking a termination codon. Science 295, 2262–2264 (2002). 11910110 10.1126/science.1067272

[b6] Tsuboi T. *et al.* Dom34:hbs1 plays a general role in quality-control systems by dissociation of a stalled ribosome at the 3′ end of aberrant mRNA. Mol Cell 46, 518–529 (2012). 22503425 10.1016/j.molcel.2012.03.013

[b7] Becker T. *et al.* Structure of the no-go mRNA decay complex Dom34-Hbs1 bound to a stalled 80S ribosome. Nat Struct Mol Biol. 18, 715–720 (2011). 21623367 10.1038/nsmb.2057

[b8] Becker T. *et al.* Structural basis of highly conserved ribosome recycling in eukaryotes and archaea. Nature 482, 501–506 (2012). 22358840 10.1038/nature10829 PMC6878762

[b9] Shoemaker C. J. & Green R. Kinetic analysis reveals the ordered coupling of translation termination and ribosome recycling in yeast. Proc Natl Acad Sci USA 108, E1392–E1398 (2011). 22143755 10.1073/pnas.1113956108 PMC3251084

[b10] Bengtson M. H. & Joazeiro C. A. P. Role of a ribosome-associated E3 ubiquitin ligase in protein quality control, Nature 467, 470–473 (2010). 20835226 10.1038/nature09371 PMC2988496

[b11] Shao S., von der Malsburg K. & Hegde R. S. Listerin-dependent nascent protein ubiquitination relies on ribosome subunit dissociation. Mol Cell 50, 637–648 (2013). 23685075 10.1016/j.molcel.2013.04.015 PMC3719020

[b12] Matsuda R., Ikeuchi K., Nomura S. & Inada T. Protein quality control systems associated with no-go and nonstop mRNA surveillance in yeast. Gene Cells 19, 1–12 (2014). 10.1111/gtc.12106 24261871

[b13] Izawa T. *et al.* Roles of Dom34:Hbs1 in nonstop protein clearance from translocators for normal organelle protein influx. Cell Rep. 2, 447–452 (2012). 22981232 10.1016/j.celrep.2012.08.010

[b14] von der Malsburg K., Shao S. & Hegde R. S. The ribosome quality control pathway can access nascent polypeptides stalled at the Sec61 translocon. Mol Biol Cell 26, 2168–2180 (2015). 25877867 10.1091/mbc.E15-01-0040 PMC4462936

[b15] Crowder J. J. *et al.* Rkr1/Ltn1 ubiquitin ligase-mediated degradation of translationally stalled endoplasmic reticulum proteins. J Biol Chem. 290, 18454–18466 (2015). 26055716 10.1074/jbc.M115.663559 PMC4513105

[b16] Burgess S. M., Delannoy M. & Jensen R. E. *MMM1* encodes mitochondrial outer membrane protein essential for establishing and maintaining the structure of yeast mitochondria. J Cell Biol. 126, 1375–1391 (1994). 8089172 10.1083/jcb.126.6.1375 PMC2290956

[b17] Orlean P., Albright C. & Robbins P. W. Cloning and sequencing of the yeast gene for dolichol phosphate mannose synthase, an essential protein. J Biol Chem. 263, 17499–17507 (1988). 3053713

[b18] Sato K. & Nakano A. Emp47 and its close homolog Emp46p have a tyrosine-containing endoplasmic reticulum exit signal and function in glycoprotein secretion in *Saccharomyces cerevisiae*. Mol Biol Cell 13, 2518–2532 (2002). 12134087 10.1091/mbc.E02-01-0027 PMC117331

[b19] Klionsky J. D. & Emr S. D. Membrane protein sorting: biosynthesis, transport and processing of yeast vacuolar alkaline phosphatase. EMBO J. 8, 2241–2250 (1989). 2676517 10.1002/j.1460-2075.1989.tb08348.x PMC401154

[b20] Green N., Fang H. & Walter P. Mutants in three novel complementation groups inhibit membrane protein insertion into and soluble protein translocation across the endoplasmic reticulum membrane of *Saccharomyces cerevisiae*. J Cell Biol. 116, 597–604 (1992). 1730771 10.1083/jcb.116.3.597 PMC2289319

[b21] Hegde R. S. & Keenan R. J. Tail-anchored membrane protein insertion into the endoplasmic reticulum. Nat Rev Mol Cell Biol. 12, 787–798 (2011). 22086371 10.1038/nrm3226 PMC3760496

[b22] Borgese N. & Fasana E. Targeting pathways of C-tail-anchored proteins. Biochim Biophys Acta 1808, 937–946 (2011). 20646998 10.1016/j.bbamem.2010.07.010

[b23] Hou B., Lin P.-J. & Johnson A. E. Membrane protein TM segments are retained at the translocon during integration until the nascent chain cues FRET-detected release into bulk lipid. Mol Cell 48, 398–408 (2012). 23022384 10.1016/j.molcel.2012.08.023 PMC3496027

[b24] Voorhees R. M. & Hegde R. S. Structure of the Sec61 channel opened by a signal sequence. Science 351, 88–91 (2016). 26721998 10.1126/science.aad4992 PMC4700591

[b25] Li L., Park W., Ling J. J., Ingram J., Ploegh H. & Rapoport T. A. Crystal structure of a substrate-engaged SecY protein-translocation channel. Nature 531, 395–399 (2016). 26950603 10.1038/nature17163 PMC4855518

[b26] Deshaies R. J. & Schekman R. A yeast mutant defective at an early stage in import of secretory protein precursors into the endoplasmic reticulum. J Cell Biol. 105, 633–645 (1987). 3305520 10.1083/jcb.105.2.633 PMC2114772

[b27] Ng D. T., Brown J. D. & Walter P. Signal sequences specify the targeting route to the endoplasmic reticulum membrane. J Cell Biol. 134, 269–278 (1996) 8707814 10.1083/jcb.134.2.269 PMC2120870

[b28] Ooi C. E. & Weiss J. Bidirectional movement of a nascent polypeptide across microsomal membranes reveals requirements for vectorial translocation of proteins. Cell 71, 87–96 (1992). 1394433 10.1016/0092-8674(92)90268-h

[b29] Yamagishi M., Fujita H., Morimoto F., Kida Y. & Sakaguchi M. A sugar chain at a specific position in the nascent polypeptide chain induces forward movement during translocation through the translocon. J Biochem. 149, 581–600 (2011). 21278156 10.1093/jb/mvr011

[b30] Kitada K., Yamaguchi E. & Arisawa M. Cloning of the *Candida glabrata TRP1* and *HIS3* genes, and construction of their disruptant strains by sequential integrative transformation. Gene. 165, 203–206 (1995). 8522176 10.1016/0378-1119(95)00552-h

