# Supplementary material for: Quality control of nonstop membrane proteins at the ER membrane and in the cytosol
Source: Sci Rep. 2016 Aug 2;6:30795. doi: 10.1038/srep30795 (PMC4969602; doi:10.1038/srep30795)
Supplement: Supplementary Information [file srep30795-s1.pdf]

Supplementary Information

Quality control of nonstop membrane proteins at the ER membrane and in the cytosol

Shunsuke Arakawa, Kaori Yunoki, Toshiaki, Izawa, Yasushi Tamura, Shuh-ichi Nishikawa, and Toshiya Endo

Contents

Figure S1. Effects of *LTN*1 deletion on cell growth upon expression of CPY-FLAG-ns and ΔSP-CPY-FLAG-ns. (**A, C**) Serial dilutions with 10-fold increments of the W303-1A (WT), *ski7*Δ and *ski7*Δ*ltn1*Δ strains with expression of CPY-FLAG or CPY-FLAG-ns (**A**) and with expression of ΔSP-CPY-FLAG or ΔSP-CPY-FLAG-ns (**C**) from the *GPD1* promoter were spotted onto SCD-Ura plates and cultivated at 23°C or 30°C for 3 or 2 days, respectively. (**B, D**) Cell extracts prepared from the indicated strains expressing CPY-FLAG or CPY-FLAG-ns (**B**) or ΔSP-CPY-FLAG or ΔSP-CPY-FLAG-ns (**D**) from the GPD1 promoter were analyzed by SDS-PAGE and immunoblotting using the indicated antibodies. The strains used for this figure were made as described in Supplemental Materials and Methods.

Figure S2. Peptidyl-tRNA is accumulated when Dom34 is absent. Whole cell extracts prepared from the indicated cells expressing the indicated nonstop proteins from the *GPD1* promoter were incubated with or without RNase A (100 μg/ml) at 37°C for 20 min, and analyzed as in Fig. 4. ns-tRNA, tRNA-attached form; ns, tRNA-dissociated form.

Supplemental Materials and Methods

The gene cassette with homologous regions to the 5' and 3'-UTR of the *LTN1* gene was amplified from pBS-natMX (*natMX;* Goldstein *et al.,* 1999) using primers ltn1-KO-F and ltn1-KO-R. The gene cassette with homologous regions to the 5' and 3'-UTR of the *SKI7* gene was amplified from pBS-hygMX (*hphMX4;* Goldstein *et al.,* 1999) using primers (ski7-KO-F: 5'-TCG TTA TTA GAG CAA TTA GCA AGA AAA AGA ATA GAA AAA TGT TGT AAA ACG ACG GCC AGT-3' and ski7-KO-R: 5'-CGA GGA ACA AAC ATG TCA AAT TCA GAG GAT GGG TGG CAA TCA CAG GAA ACA GCT ATG ACC-3'). The amplified fragments were introduced into W303-1A (*MATa ade2-1 his3-11,15 ura3-1 leu2-3,112 trp1-1 can1-100*) to obtain *ski7*Δ or *ski7*Δ*ltn1*Δ strains. Strains expressing C-terminally FLAG-tagged CPY, CPY-ns or ΔSP-CPY-ns, were generated by transforming W303-1A, *ski7*Δ or *ski7*Δ*ltn1*Δ with pIZ108, pIZ106 or pIZ107, respectively[13]. To express C-terminally FLAG-tagged ΔSP-CPY, a DNA fragment corresponding to CPY ORF lacking residues 2-20 was amplified by PCR, and inserted into the *Spe*I and *Eco*RI sites of pIZ7.

Supplemental Reference

Goldstein, A. L., and McCusker, J. H. Three new dominant drug resistance cassettes for gene disruption in *Saccharomyces cerevisiae*. *Yeast* **15**, 1541–1553 (1999).

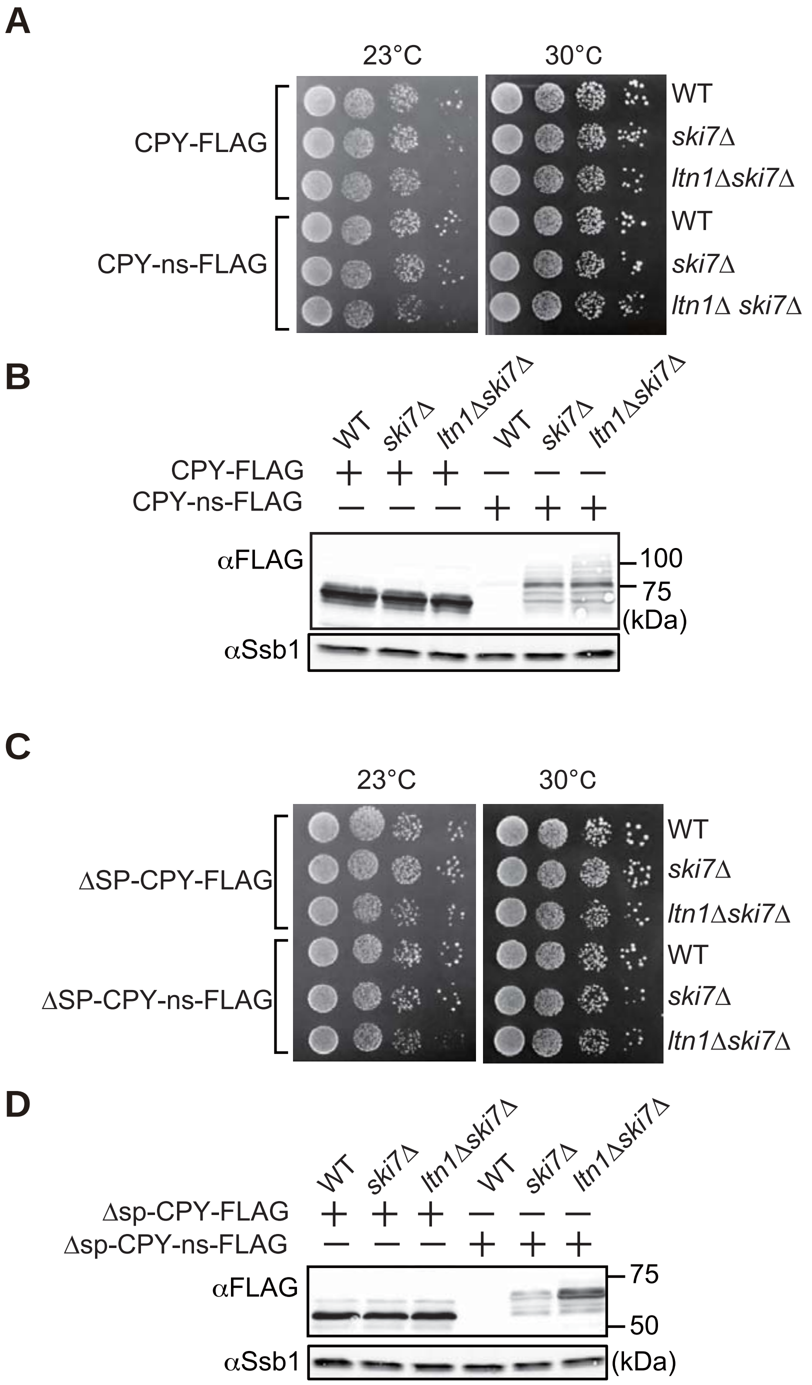

Arakawa et al. Figure S1

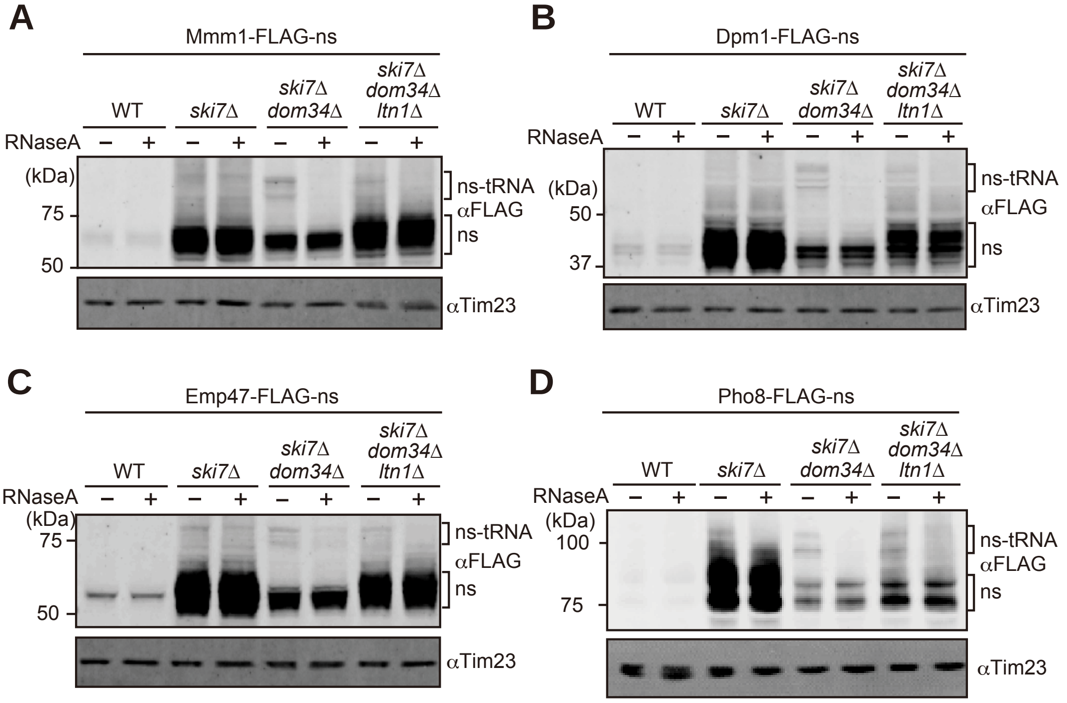

Arakawa et al. Figure S2
